# Geospatial clustering of type 1 diabetes in Sweden: a cohort study based on all residential locations from birth to diagnosis

**DOI:** 10.1007/s00125-026-06675-9

**Published:** 2026-02-16

**Authors:** Samy Sebraoui, Oskar Englund, Fredrik Nyberg, Annelie Carlsson, Olle Korsgren, Gun Forsander, Katarina Eeg-Olofsson, Björn Eliasson, Hanne K. Carlsen, Karin Åkesson, Soffia Gudbjörnsdottir

**Affiliations:** 1 Department of Molecular and Clinical Medicine, Sahlgrenska Academy, University of Gothenburg, Gothenburg, Sweden; 2 Department of Natural Sciences, Design and Sustainable Development, Mid-Sweden University, Östersund Campus, Sweden; 3 School of Public Health and Community Medicine, Institute of Medicine, Sahlgrenska Academy, University of Gothenburg, Gothenburg, Sweden; 4 Lund University Diabetes Centre, Department of Clinical Sciences, Skåne University Hospital, Lund University, Lund, Sweden; 5 Department of Immunology, Genetics and Pathology, Uppsala University, Uppsala, Sweden; 6 Department of Paediatrics, Institute of Clinical Sciences, Sahlgrenska Academy, University of Gothenburg, Gothenburg, Sweden; 7 Department of Endocrinology and Metabolism, Sahlgrenska University Hospital, Gothenburg, Sweden; 8 The Swedish National Diabetes Register, Centre of Registers, Gothenburg, Sweden; 9 Department of Paediatrics, Ryhov County Hospital, Jönköping, Sweden

**Keywords:** Disease mapping, Environmental exposures, Environment-wide association study, Epidemiology, Geographical variation, Incidence, Risk factors, Space-time clustering, Type 1 diabetes

## Abstract

**Aims/hypothesis:**

Type 1 diabetes develops gradually, and previous exposures may influence incidence. We aimed to assess the geographical variation in type 1 diabetes incidence in Sweden by considering all residential locations from birth to diagnosis in individuals aged 0–30 years, diagnosed between 2005 and 2022. Significant high- and low-risk clusters were identified for different life stage exposure windows.

**Methods:**

In 21,774 individuals with type 1 diabetes, all residential geographical locations from birth to diagnosis were geocoded. Geostatistical analysis of the incidence of type 1 diabetes was conducted at the municipality level using the most common residential location during four life stage-specific exposure windows (at diagnosis, the first 5 years after birth, 5 years prior to diagnosis, and from birth to diagnosis). Spatial scan statistics were used to identify statistically significant high- and low-risk clusters for each window. Land use and land cover within these clusters were also characterised.

**Results:**

Significant geographical variation in the incidence of type 1 diabetes was observed. The incidence was consistently higher in rural, low-population-density areas, particularly in central Sweden, and lower in major urban areas. The largest number of spatial clusters of both high risk (RR 1.29–16.0) and low risk (RR 0.32–0.73) was identified when using the most common residential location during the first 5 years after birth. High-risk clusters for this exposure window were characterised by forested and agricultural land, while low-risk clusters were characterised by urban land and open land other than agricultural land.

**Conclusions/interpretation:**

Our findings suggest that the development of type 1 diabetes in Sweden varies geographically and is associated with specific features of the local surroundings in early childhood. This is important knowledge as a basis for identifying possible environmental risk factors and the relationship with risk of type 1 diabetes in future studies.

**Graphical Abstract:**

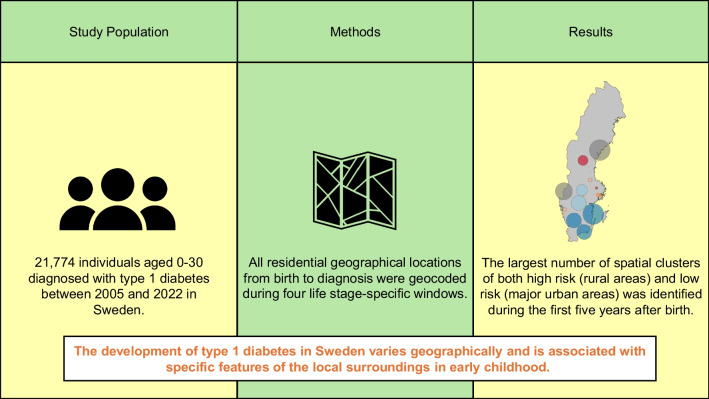

**Supplementary Information:**

The online version contains peer-reviewed but unedited supplementary material available at 10.1007/s00125-026-06675-9.


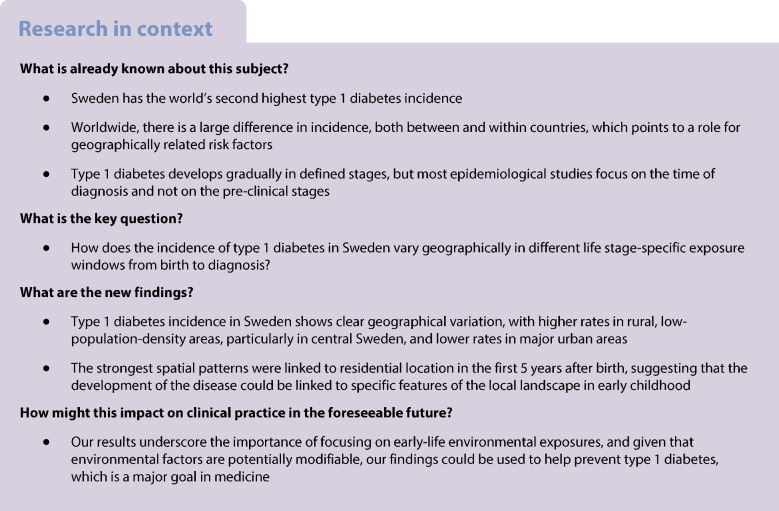


## Introduction

Type 1 diabetes is an autoimmune disorder marked by elevated blood glucose levels that results from an insulin deficiency caused by destruction of pancreatic islet beta cells [1]. It is the most common serious chronic disease of childhood. The incidence rate peaks at the age of 10–14 years; however, it can develop at any age and 50% of patients are diagnosed as adults [2, 3]. Individuals with type 1 diabetes need lifelong insulin treatment to survive. Although the management of the disease and survival rates have improved dramatically in recent years, mortality is still twofold to eightfold higher, and life expectancy in individuals who develop the disease by age 20 years is approximately 12 years lower than that in a non-diabetic population [4].

Sweden has the second highest incidence of type 1 diabetes in the world [5]. Both in Sweden and worldwide, the incidence has increased by approximately 3–4% per year [2, 6, 7]. This rapid increase cannot be explained by genetic susceptibility. Furthermore, there is wide variation in incidence both between and within countries, which suggests that differences in environmental factors may influence the risk of the disease [1, 8]. Potential environmental triggers include in-utero exposures, perinatal triggers, viral infections, dietary components, and alterations in the gut microbiome [8, 9]. Moreover, while research in this area remains limited, geographical location and geographically linked environmental factors are hypothesised to play a crucial role [10].

The pathogenesis of type 1 diabetes is now recognised as a continuum that might take years to clinical diagnosis and can be characterised by three stages: (1) detection of at least two autoantibodies in the absence of hyperglycaemia; (2) development of hyperglycaemia; and (3) development of overt diabetes [11]. Previous studies on geographical variation and possible environmental triggers have focused solely on the time of diagnosis and not on the earlier stage of the disease [10, 12–17]. Studying longitudinal exposure across different time windows could capture critical periods of the development stages of the disease and add important knowledge underlying the pathogenesis of type 1 diabetes.

Using spatially explicit data on both cases and the total population, geographical high- and low-risk clusters can be identified using spatial scan statistics [18]. To study geographical variation in detail requires advanced use of Geographic information systems (GIS) [19–21]. Furthermore, studying geographical variation for different life stages requires spatial data on each residential location of every individual, from birth to diagnosis. However, access to such data in many countries is often limited. Custom GIS models can be developed to compute geostatistical correlations between disease incidence and potential environmental determinants [19]. This method offers a novel opportunity to uncover previously unrecognised risk factors and holds potential for broader applications across various domains of medical research.

The aim of this study was to examine the geographical variation in type 1 diabetes incidence in Sweden by accounting for the geographical location from birth to diagnosis for all new cases diagnosed at the age of 0–30 years during the years 2005 to 2022. Geostatistical high-risk and low-risk clusters were computed for different life stages leading up to the diagnosis.

## Methods

### Data sources

This study used data from the Swedish National Diabetes Register (NDR), a nationwide quality register established in 1996 that contains comprehensive clinical information, diabetes type, date of diagnosis, complications and treatments. Inclusion in the NDR requires informed consent, either oral or written. The registry achieves near-complete national coverage, capturing approximately 98% of children and approximately 90–95% of adults with type 1 diabetes in Sweden*,* with no systematic geographical differences [22].

Individuals included in this study were diagnosed with type 1 diabetes at the age of 0–30 years and with the presence of at least one recorded observation in NDR between 1 January 2005 and 31 December 2022. The inclusion criterion was defined as a clinical definition of type 1 diabetes [23] including latent autoimmune diabetes in adults. We first included 22,005 patients; 202 patients were excluded due to missing data on geographical coordinates and 29 patients were excluded from the final cohort due to changed diagnosis to type 2 diabetes, other specific types of diabetes or unclear clinical classification. The final cohort included 21,774 patients. This study included both female and male individuals with type 1 diabetes, with sex ascertained from personal identification numbers. Gender identity was not recorded.

By linking NDR with other databases, which is possible to achieve with very high accuracy due to the Swedish system with unique personal identification numbers, Statistics Sweden (SCB) identified all residential geographical locations for each case with type 1 diabetes for each year from birth to diagnosis, sex, year of birth as well as country of birth for all cases and for both parents. The personal identification number from NDR was de-identified by SCB and replaced with a pseudonym.

Residential location data were geocoded using official address coordinates provided by SCB; these are accurate to the property or dwelling level with a precision of one metre. Geospatial coordinates were further processed using Quantum GIS (QGIS, version 3.40.6) [24], with all coordinates projected in Swedish Reference Frame 1999, Transverse Mercator (SWEREF 99 TM) [25]. Several adjustments were made to the population datasets to enable different analyses, as detailed below. The analysis was divided into four parts.

The background population was provided by SCB for the same age group for the whole study period.

### Life-stage-specific exposure windows

All analyses in this study considered all the residential locations of all cases from birth to diagnosis. Four life stage-specific exposure windows were defined: (1) the residential location of each case in the year of diagnosis; (2) the most dominant residential location of each case during the first 5 years after birth; (3) the most dominant residential location of each case during the 5 years prior to diagnosis; and (4) the most dominant residential location of each case from birth to diagnosis.

Four vector (point) datasets were developed according to the four life stage residential locations by first identifying coordinates from the case database and then converting them into geospatial data in QGIS as stated above.

### Incidence analysis at the municipality level

We conducted a geostatistical analysis of the incidence of type 1 diabetes in each of the 290 municipalities of Sweden for each life stage-specific exposure window.

The mean background population in the age group 0–30 years in each municipality during the study period 2005–2022 was calculated.

We used standard geospatial algorithms in QGIS (‘count points in polygons’) to calculate the number of cases in each municipality for the four life stage exposure window datasets. These numbers were then compared with the mean background population to calculate crude incidence. We also calculated the difference between observed cases and expected cases, i.e. the product of municipality population, national incidence and time period in each municipality.

In addition to visualising the municipality results in an actual map of Sweden with municipal borders, a Dorling cartogram [26] was computed to enable visual comparison of incidence relative to total population density for each municipality. This allowed us to illustrate the municipalities as circles where the size of the circle is proportional to the municipality population.

**Fig. 1:**
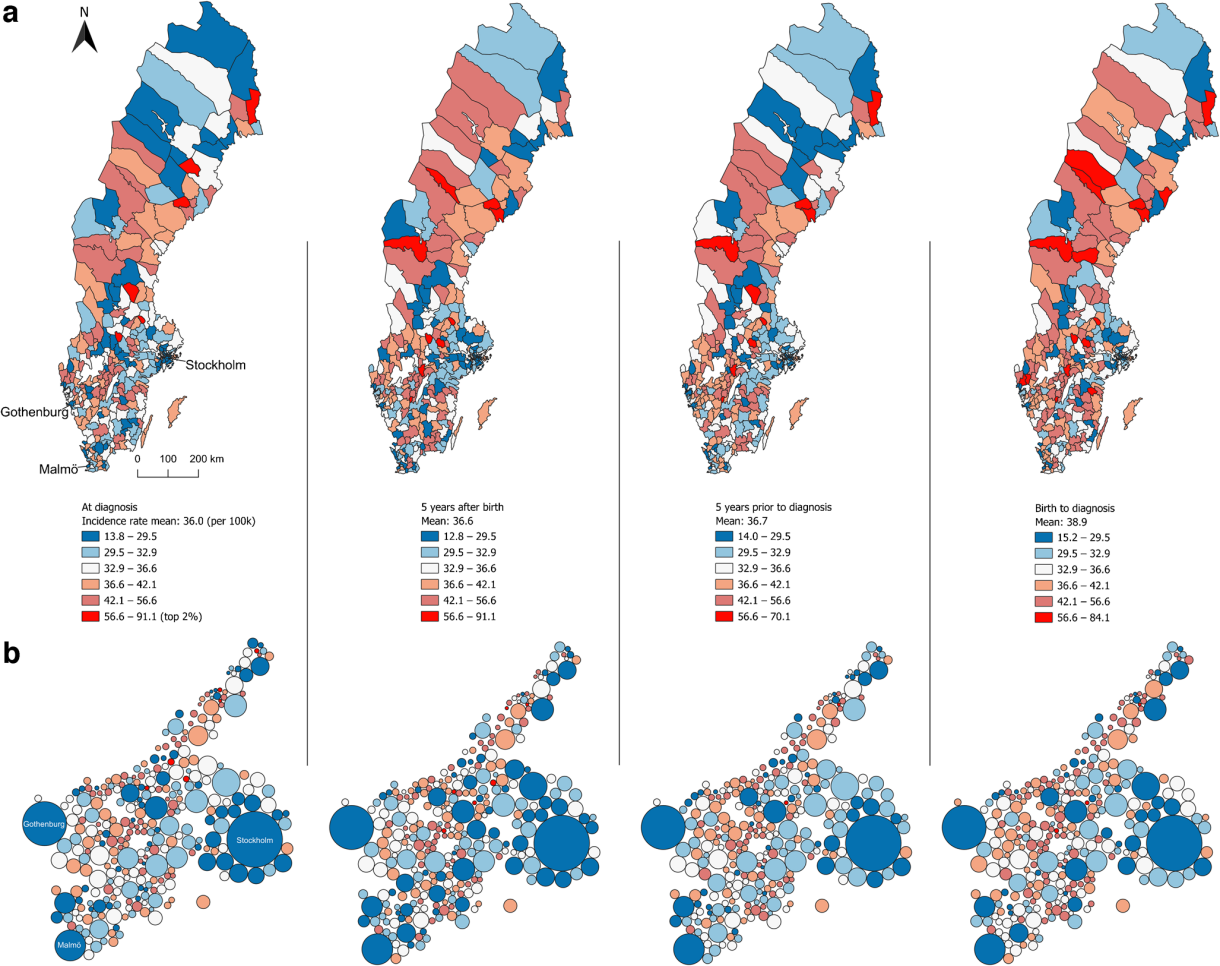
Geographical distribution at the municipality level for four different life stage-specific exposure windows of the incidence of type 1 diabetes in individuals diagnosed in Sweden between 2005 and 2022 at the age of 0–30 years. Displayed in quintiles (coloured blue to orange) and the top 2% of the highest rate (red). (**a**) Crude incidence of type 1 diabetes (cases per 100,000 person-years). (**b**) The same information as in (**a**) illustrated in a Dorling cartogram; municipalities presented as circles; the circle size is proportional to the municipality total population

**Fig. 2:**
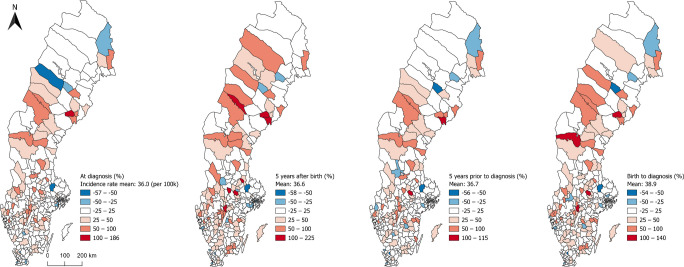
Geographical distribution at the municipality level for four different life stage-specific exposure windows of the difference between the observed and expected number of cases (%) of type 1 diabetes in individuals diagnosed in Sweden between 2005 and 2022 at the age of 0–30 years. Displayed in fixed intervals representing percentage differences (coloured blue to red)

To assess the influence of genetic and cultural backgrounds on the incidence of type 1 diabetes, the analyses were repeated after stratification into two different ethnic groups: (1) individuals born in Sweden with two Swedish-born parents (natives); and (2) individuals not born in Sweden or Sweden-born with at least one non-native parent (non-natives) (electronic supplementary material [ESM] Figs 1, 2).

### Spatial scan cluster analysis

For each life stage residential location, we identified statistically significant geographical high- and low-risk clusters across the country independent from artificial boundaries such as municipalities using the discrete Poisson regression model in software for the spatial, temporal and space-time scan statistics (SaTScan) version 10.2.525 [27]. Official population data from SCB were used to calculate the mean background population in the age group 0–30 years during the study period, with a 1×1 km spatial resolution, which served as the denominator for each grid cell. After excluding cells with zero inhabitants, a mean of 104,540 cells remained. Each case was geocoded to a population cell and the cell was spatially represented by its centroid, the geometric centre of a shape or object, which allows for irregularly shaped clusters.

The significance of the clusters was assessed through 999 Monte Carlo simulations, comparing the results with the actual geographical distribution of cases with the maximum likelihoods from each of the random replicas of the datasets [18]. The maximum population at risk threshold was set to 5% and the maximum cluster radius to 100 km. These parameters were chosen to prioritise the detection of smaller, epidemiologically relevant clusters while avoiding large, heterogeneous regions characterised by population density gradients [28]. The ‘population at risk’ here only refers to the maximum proportion of the total population that can be included within a cluster. The null hypothesis of complete spatial randomness was rejected at the significance level of *p*≤0.05. A statistically significant high-incidence cluster was interpreted as an area within a circular scanning window exhibiting a higher RR [18, 28, 29] of type 1 diabetes compared with the rest of the study region (i.e. the population outside the cluster).

### Land use and land cover analysis

To explore environmental characteristics associated with the risk of type 1 diabetes, we examined land use and land cover (LULC) within statistically significant high- and low-risk clusters identified by SaTScan in part 3 of the analysis. The significant clusters were compared with the official land-use map for Sweden [30] using the QGIS tool Zonal Histogram [24]. The original 17 LULC classes were combined into five main LULC classes: (1) ‘Urban’, including the LULC classes buildings, other built-up land and roads/railways; (2) ‘Agriculture’, including all agricultural land; (3) ‘Forest’, including all forest categories; (4) ‘Open land’, including wetlands and other open land with and without vegetation; and (5) ‘Lakes and streams’, including lakes and streams. The distribution of LULC classes in high- and low-risk areas were then compared to assess environmental patterns potentially related to type 1 diabetes risk. The RR associated with different LULC characteristics such as ‘lake and stream’ or ‘forest’ areas refers to the population residing in areas characterised by such land cover, not to the water bodies or forests themselves.

### Ethics

This study was approved by the Swedish Ethical Review Authority, permit number 2022–02866–01, and was carried out in accordance with the EU’s General Data Protection Regulation rules.

## Results

A total of 21,774 individuals aged 0–30 years were diagnosed and recorded in NDR with type 1 diabetes in Sweden between 2005 and 2022 (Table 1). The mean age at diagnosis was 13.6 years (14.1 years for boys and 12.9 years for girls); 57.7% of participants were male. The mean±SD age at diagnosis decreased from 13.6±7.2 in 2005 to 12.0±7.2 years in 2022. A total of 15,426 (70.8%) individuals were younger than 18 years at the time of diagnosis. Overall, 16,590 (76.2%) were born in Sweden to two Swedish-born parents, while 5178 (23.8%) were not born in Sweden or Sweden-born with at least one non-native parent.

**Table 1:** Demographics of the participants grouped by sex and age groups at diagnosis

Age at diagnosis	Total *N*=21,774	Male *N*=12,556 (57.7)	Female *N*=9218 (42.3)
0–4 years	2604 (12.0)	1426 (11.4)	1178 (12.8)
5–9 years	4688 (21.5)	2419 (19.3)	2269 (24.6)
10–14 years	5726 (26.3)	3192 (25.4)	2534 (27.5)
15–19 years	3592 (16.5)	2354 (18.7)	1238 (13.4)
20–24 years	2494 (11.5)	1499 (11.9)	995 (10.8)
25–30 years	2670 (12.3)	1666 (13.3)	1004 (10.9)

Data are *n* (%)

### Incidence at the municipality level

The mean national incidence was 36.0 per 100,000 person-years over the entire study period. The incidence increased by 2.8%, from 35.6 per 100,000 person-years in the period 2005–2010 to 36.6 per 100,000 person-years in the period 2016–2022. The incidence of type 1 diabetes during 2005–2022 per municipality varied from 13.8 to 91.1 per 100,000 person-years. Higher incidence was observed in central areas and parts of northern Sweden and lower incidence was consistently seen in municipalities with large cities. The Dorling cartogram illustrates this pattern clearly (Fig. 1a, b).

Approximately 24% of all individuals had relocated from their municipality of birth by the time of diagnosis, with a notable effect on incidence patterns when analysing the four different life stage exposure windows. While some municipalities had relatively high or low incidence for all life stage windows, others changed notably. However, the low incidence in municipalities with large cities was consistent for all life stage windows. The greatest difference in spatial incidence patterns was seen during the first 5 years after birth (Fig. 1a).

By visualising the difference between observed and expected number of cases, municipalities around major cities consistently exhibited fewer than expected cases while some central areas in the country consistently showed more than expected cases (Fig. 2). The geographical distribution at the time of diagnosis mostly resembled the results for the period 5 years prior to diagnosis, and the geographical distribution for the first 5 years after birth resembled more closely the results between birth and diagnosis. It should be noted that municipalities with high incidence are sometimes adjacent to municipalities with low incidence, indicating that actual geographical patterns may be obscured by artificial administrative boundaries.

When the analysis was repeated using data only from individuals born in Sweden with two Sweden-born parents, similar geographical patterns were observed (ESM Fig. 1), with lower incidence in the largest cities. At the time of diagnosis, the incidence varied from 13.3 to 106.5 per 100,000 person-years across the municipalities. The mean incidence in this subgroup was 41.2 per 100,000 person-years (vs 36.0 per 100,000 person-years when including all cases). When using data from the first 5 years after birth in this subgroup, numerous municipalities across the country exhibited a relatively high incidence, particularly in the central region. Municipalities that demonstrated low incidence when all cases were included, particularly in the southern regions of the country (Fig. 1a), showed markedly higher incidence in this subgroup (ESM Fig. 1a).

### High- and low-risk spatial clusters

**Fig. 3:**
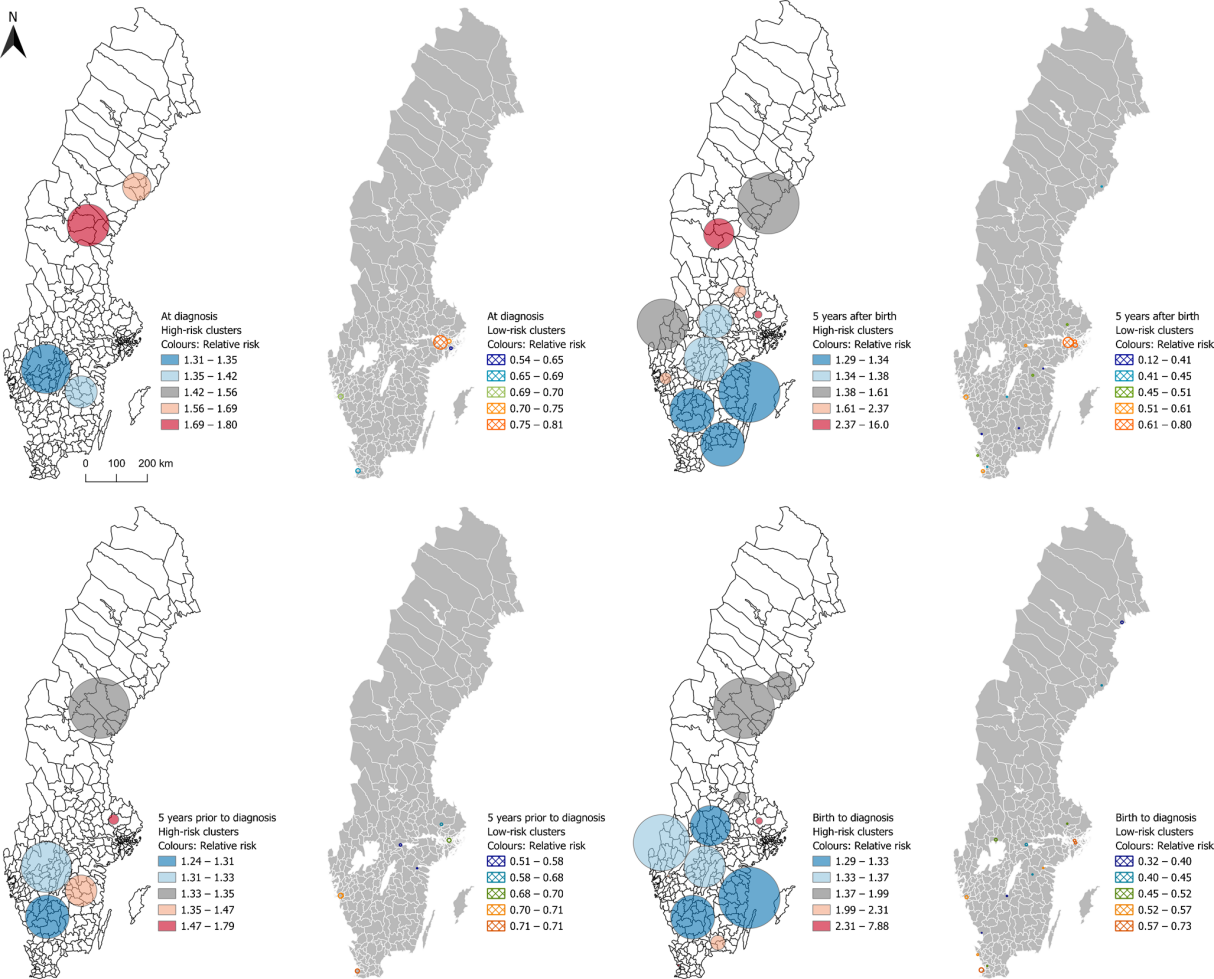
Statistically significant high- and low-risk clusters of type 1 diabetes for four different life stage-specific exposure windows, identified using spatial scan statistics on data from individuals diagnosed in Sweden between 2005 and 2022 at the age of 0–30 years. The circles represent the actual geographical regions covered by the identified clusters. Displayed in quintiles (coloured blue to orange). Two very small clusters for the exposure windows ‘5 years after birth’ with RR 16.0 in mid-east Sweden and ‘birth to diagnosis’ with RR 7.88 in west Sweden have been removed from the map to protect patient confidentiality

When using data on the residential location at diagnosis, several significant high-risk clusters were identified across Sweden, with RR ranging from 1.31 to 1.80 (Fig. 3). The largest cluster was identified between the two largest lakes located in the middle of the country, and the highest RR was in northern Sweden. Significant low-risk areas were consistently identified around major cities.

Data on the residence in the first 5 years after birth generated the largest number of clusters, identifying high-risk areas along the southeastern coast with RR ranging from 1.29 to 1.34. The second most northern cluster had an RR of 2.73 and 41 cases in a population of 2647. The cluster with the highest number of cases (966 in a population of 125,650) was located between the two largest lakes with an RR of 1.37. Smaller high-risk clusters appeared near Gothenburg and Stockholm. One small cluster with an exceptionally high RR of 16.00 was observed in mid-east Sweden (8 cases in a population of 88). This cluster has been removed from the map to protect patient confidentiality (Fig. 3).

When using data from the 5 years prior to diagnosis, a new cluster emerged in southern Sweden with an RR of 1.24, while the cluster in northern Sweden expanded and its RR was reduced from 1.80 to 1.35, compared with clusters using data on the residential location at diagnosis (Fig. 3).

Using data from birth to diagnosis, 13 significant high-risk clusters of varying size were identified, with RR ranging from 1.23 to 2.51. One small but high-risk cluster had an exceptionally high RR of 7.88 (13 cases in a total population of 270) located in west Sweden. This cluster has been removed from the map to protect patient confidentiality*.* No high-risk clusters were observed in major cities. Several high-risk clusters were identified in southern Sweden. The cluster with the highest number of cases (818 in a population of 100,255) was located between the two largest lakes. Low-risk clusters were consistently concentrated around major cities (Fig. 3 and ESM Fig. 3).

### Land cover characteristics of high- and low-risk clusters

**Fig. 4:**
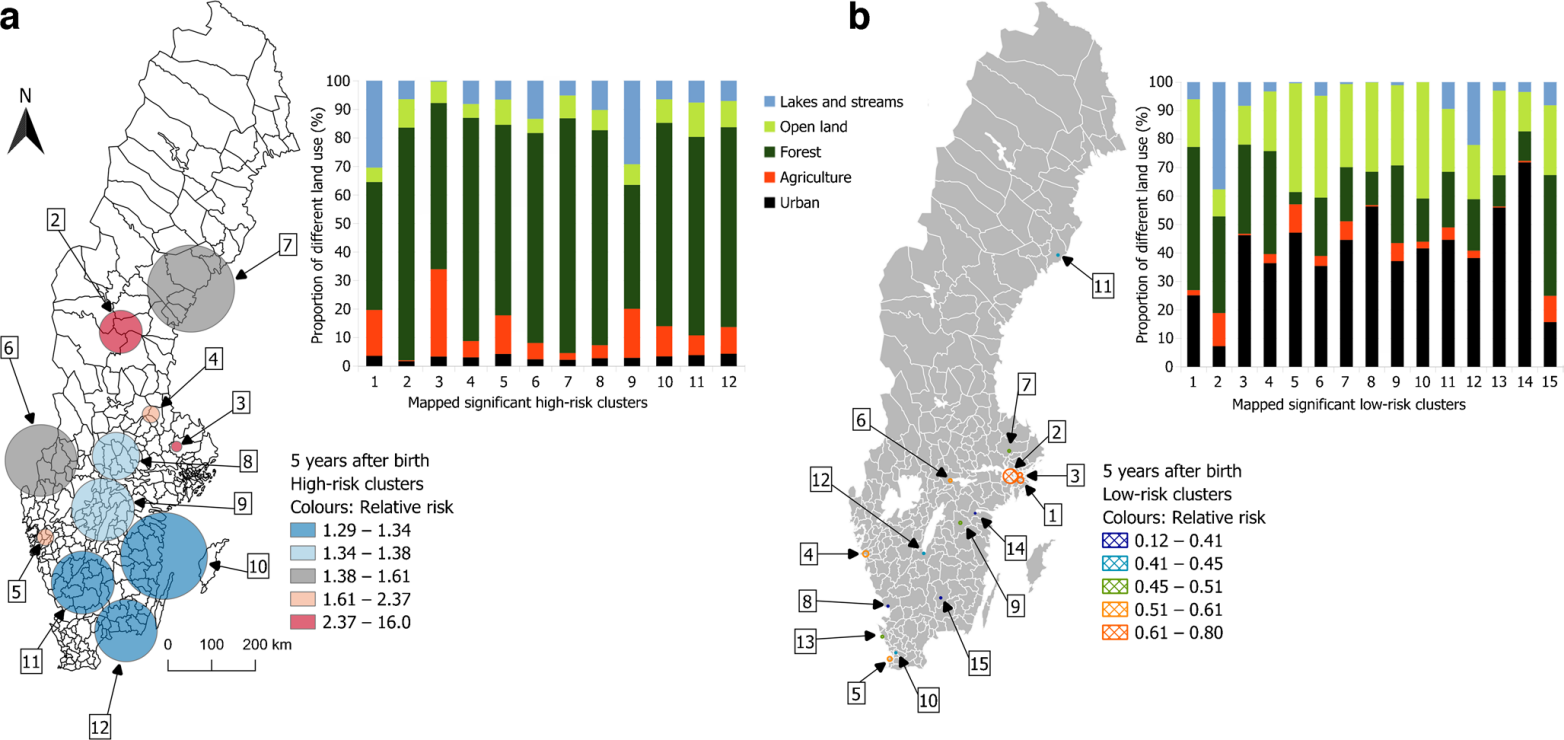
Proportion of different land uses in significant high- and low-risk clusters using the most common location in the first 5 years after birth, from individuals diagnosed in Sweden between 2005 and 2022 at the age of 0–30 years, sorted by RR in a descending order, starting with the highest RR. The circles represent the actual geographical regions covered by the identified clusters. (**a**) High-risk clusters and (**b**) low-risk clusters. One very small high-risk cluster (number 1) has been removed from the map to protect patient confidentiality. All low-risk clusters are located in the largest cities

**Fig. 5:**
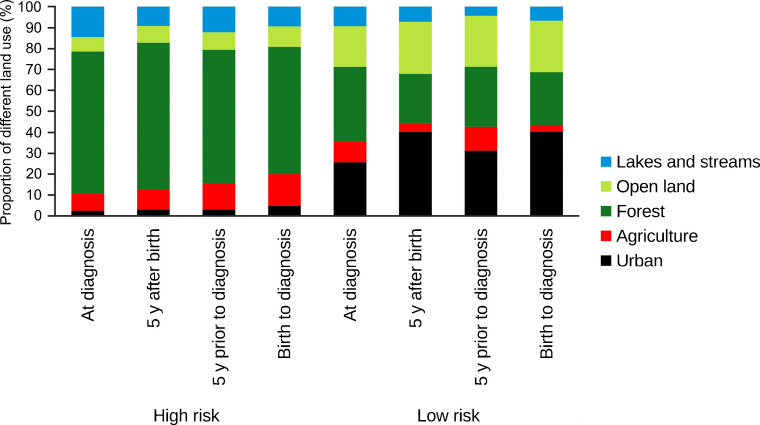
Proportion of different LULC classes in significant high- and low-risk clusters for type 1 diabetes. Mean share of LULC classes within each of the four life stage exposure windows. All low-risk clusters are located in the largest cities. y, years

A clear distinction was observed between high- and low-risk clusters in terms of LULC composition. High-risk clusters were predominantly characterised by forested areas and a relatively high proportion of agricultural land, whereas low-risk clusters were mainly composed of urban land and open land other than agriculture. This pattern was consistent both within and between all four life stage exposure windows (Figs 4, 5).

## Discussion

In this nationwide study of individuals diagnosed with type 1 diabetes in Sweden between 2005 and 2022 at the age of 0–30 years, we identified a notable geographical variation in incidence. Using high-resolution geospatial analyses, we observed that the incidence of type 1 diabetes was consistently higher in rural and low-population-density areas particularly in the central part of the country and lower in major urban areas. These patterns were consistent using various analytical approaches and between four different life stage-specific exposure windows. The most striking findings emerged when evaluating the most dominant residential location in the first 5 years after birth, which revealed the highest number of significant high-risk and low-risk spatial clusters.

Nearly one in four individuals in this study relocated between municipalities between birth and diagnosis, underscoring the potential for exposure misinterpretation in studies relying solely on residential location at the time of diagnosis or even later [10, 13, 14, 17]. By incorporating longitudinal residential data and analysing different life stage exposure windows, our approach captures critical periods of the development of the disease, including early-life exposure, which may contribute differently to type 1 diabetes pathogenesis. Patients in the high-risk clusters were less likely to relocate to a different municipality compared with those in the low-risk clusters, both from birth to diagnosis (26.4% vs 38.7%) and during the first 5 years after birth (25.1% vs 28.4%). This may have contributed to the stronger association between place of birth and subsequent incidence.

Across the 290 municipalities the incidence rate varied more than sixfold from 13.8 to 91.1 per 100,000 person-years. The highest incidence was found predominantly in sparsely populated municipalities located in central Sweden, without a clear north-south gradient, in contrast to the findings of previous studies [31]. Conversely, the lowest risk was observed in urban areas around the largest cities. These results are in line with previous data from the Nordic countries of children 0–14 years old during 2006–2011 [32], as well as with an older study from Finland [33]. Our study extends these findings across a wider age span and over a longer period. Most importantly, our findings suggest that the risk associated with geographical location is more pronounced when considering early-life residential location rather than location at diagnosis.

Both the risk of type 1 diabetes and the residential distribution of individuals with non-Swedish ethnicity vary across the country. A previous study showed that children in families that have immigrated to Sweden have lower incidence compared with Sweden-born children [34]. These factors cannot explain our findings since separate analysis for individuals born in Sweden to two Sweden-born parents showed the same general geographical patterns for all life stage exposure windows when compared with the analysis with all cases included. However, municipalities that previously demonstrated low incidence when all cases were included, particularly in the southern regions of the country, showed higher incidence when the non-native group was excluded. This phenomenon could be explained by immigration.

The study of geographical variation based on municipalities is limited as some municipalities are very sparsely populated and only a few cases might make a large difference, and the large number of municipalities implies a risk of significance by chance. In addition, artificial boundaries that do not account for geographical or environmental factors may result in overlooking accumulations of geographical influences that are spread across multiple municipalities. To overcome these limitations, we also identified clusters independently of any artificial boundaries. This approach provided a more detailed analysis compared with methods based on municipal boundaries and should result in more accurate geographical patterns.

High-risk and low-risk clusters were analysed for the four life stage exposure windows. Again, this approach is essential given that the disease takes years to develop [11] and that one fourth of all individuals relocated between birth and diagnosis. However, no earlier studies have assessed geographical exposure at different life stages leading up to the development of the disease.

The cluster analysis at diagnosis revealed four significant high-risk clusters with an RR from 1.31 to 1.80 located in the mid country, and five low-risk clusters in the largest cities, which largely mirrored the results from the municipality-based analysis. Country-specific incidence rate studies have shown rural excess in some countries of type 1 diabetes [10, 12, 15, 16, 35] but not all [13, 36–38]. These studies vary in terms of methodology, and some studies only have a few cases and small geographical units. The characteristics of rural/urban environments also need to be considered. Several studies have found a higher incidence of type 1 diabetes in areas with lower levels of deprivation [35, 39–42]. A study from Sweden has shown that low maternal education increases the risk of the disease [43]. In the current study, the observed differences in incidence between rural and urban areas could be attributed to spatial patterns in the population composition of individual-level socioeconomic status, which may be related to differences in lifestyle, exposure to infections or other exposures.

The LULC analyses revealed a distinct urban–rural contrast: high-risk clusters were largely in rural areas, with a high proportion of forested and agricultural land; low-risk clusters were dominated by urban land and open land other than agriculture. Given that the highest number of significant high-risk and low-risk spatial clusters were found for the first 5 years after birth, this suggests that the risk for type 1 diabetes could be linked to specific features of the local surroundings in early childhood, independently of the age of diagnoses. These could be associated with environmental risk factors specific to rural living or protective factors in urban living during early childhood. Our results are consistent with growing evidence suggesting that early-life events including parental psychosocial stress, pregnancy-related factors, infant growth patterns, infections, and environmental exposures may influence the later development of type 1 diabetes [8, 9, 44, 45]. This is important knowledge to be able to identify possible early environmental risk factors of type 1 diabetes in future studies. Our finding of lower risk in urban areas is in line with a recent study from England, which reported that air pollution, light at night, population density, and overcrowding were negatively associated with type 1 diabetes incidence [10].

Furthermore, day care attendance may be associated with a reduced risk of type 1 diabetes [46]. Our findings could imply the presence of a protective effect linked to characteristics of the urban environment and might partly be explained by the hygiene hypothesis, which posits that children with low exposure to infectious agents at an early stage after birth have an increased susceptibility for type 1 diabetes [44, 47–49].

It is unlikely that a difference in geographical distribution of high-risk HLA haplotypes could explain our finding, especially given that less than 5% of individuals with these haplotypes in the general population develop overt type 1 diabetes, strongly suggesting a second critical hit is needed [50].

The strengths of this study are that it is a large nationwide study from a country with a high incidence of type 1 diabetes, covering both children and young adults. We used precise geographical coordinates of all cases from birth to the year of diagnosis. High-resolution geospatial analyses were used to identify geographical incidence patterns during different life stage exposure windows. This is unique and has not been previously conducted in diabetes epidemiology. All analyses were compared with those of the mean population in the same age group, during the study period, and thus, the individuals with type 1 diabetes were consistently matched with those in the corresponding population group. We have also quantitatively characterised all high- and low-risk clusters in terms of land use/land cover.

The weaknesses of the study are that spatial analysis is sensitive to sample size, which implies that areas with a small number of type 1 diabetes cases may result in the identification of large areas as ‘hot spots’ or clusters. Given the large study population and the long observation time, this should not affect our conclusions. The coverage of NDR was somewhat lower during the first 2 years of the study period. However, NDR collects repeated registrations (including date or year of diagnosis) at least annually; therefore, almost all patients with type 1 diabetes diagnosed between 2005 and 2022 are eventually captured. We lacked access to gridded population data for the period 2005–2014. Consequently, it was necessary to interpolate population estimates based on municipal population data from 2015–2022, which were subsequently transferred to the grid cells. Municipal population data over time exhibited minimal variation. Type 1 diabetes is a relatively rare disease, and robust cluster analyses require large patient numbers to ensure statistical certainty. Subdivisions in time periods or age groups further reduce numbers and power; thus, such data could be prone to spurious findings. Furthermore, we did not assess whether the results differed between male and female individuals or explore gender-related factors, which limits generalisability across all sexes and genders.

We deliberately chose to use crude incidence rate to ensure methodological consistency and comparability across the difference analyses. Sensitivity analyses did not show any substantial differences between age- and sex-standardised incidence rates and crude incidence. Thus, the methodical choice is highly unlikely to affect our conclusions (ESM Fig. 4).

### Conclusion

Type 1 diabetes incidence in Sweden shows clear geographical variation, with higher rates in rural, low-population-density areas, particularly in central Sweden, and lower rates in major urban areas. The strongest spatial patterns were linked to residence in early childhood, suggesting that local environmental features – such as forested and agricultural land – may influence disease risk. These findings underscore the importance of investigating early-life environmental exposures in future type 1 diabetes research.

## Supplementary Information

Below is the link to the electronic supplementary material.

ESM Figures (PDF 1258 KB)

## Data Availability

The datasets analysed during the current study are not publicly available as they contain information about individual cases. The population datasets provided by SCB are available online in open-access: SCB population statistics, SCB municipality level, SCB grid cells.

## Abbreviations

GIS: Geographic information system
LULC: Land use and land cover
NDR: National Diabetes Register
QGIS: Quantum geographic information system
SaTScan: Software for the spatial, temporal and space-time scan statistics
SCB: Statistics Sweden
SWEREF 99 TM: Swedish Reference Frame 1999, Transverse Mercator
