# Supplementary material for: Geospatial clustering of type 1 diabetes in Sweden: a cohort study based on all residential locations from birth to diagnosis
Source: Diabetologia. 2026 Feb 16;69(5):1237–48. doi: 10.1007/s00125-026-06675-9 (PMC13005807; doi:10.1007/s00125-026-06675-9)
Supplement: Supplementary file 1 — ESM Figures (PDF 1258 KB) [file 125_2026_6675_MOESM1_ESM.pdf]

Electronic supplementary material (ESM)

Title: Geospatial clustering of type 1 diabetes in Sweden: a cohort study based on all residential locations from birth to diagnosis

Authors: Samy Sebraoui, Oskar Englund, Fredrik Nyberg, Annelie Carlsson, Olle Korsgren, Gun Forsander, Katarina Eeg-Olofsson, Björn Eliasson, Hanne K Carlsen, Karin Åkesson and Soffia Gudbjörnsdottir

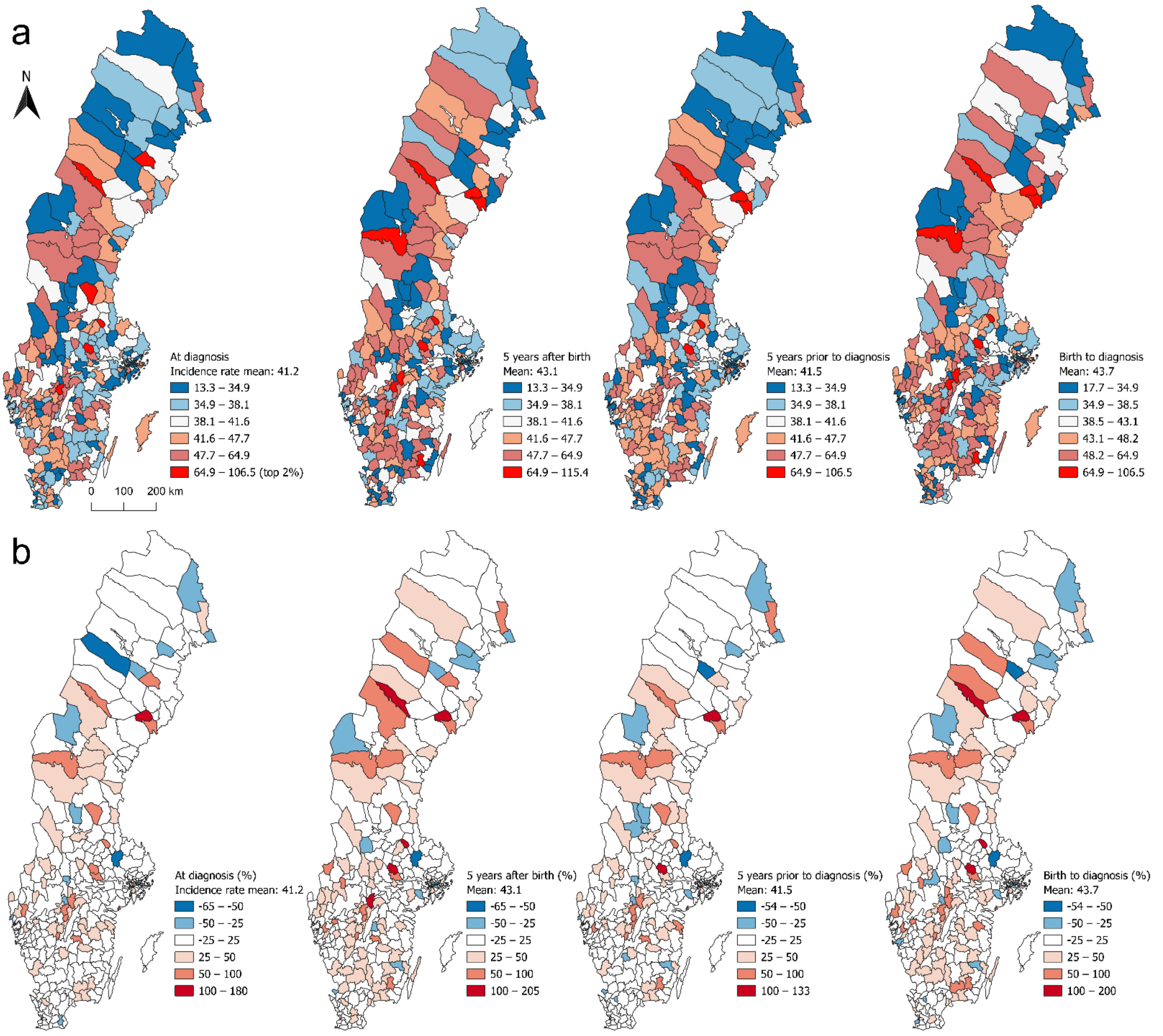

ESM Fig. 1: Geographical distribution at the municipality level for four different life stage-specific exposure windows of the incidence of type 1 diabetes in native individuals diagnosed in Sweden between 2005 and 2022 at the age of 0-30 years. a) Crude incidence of type 1 diabetes (cases per 100,000 person-years) by native/non-native background. Displayed in quintiles and the top 2% of the highest rates; b) Differences (%) between observed and expected numbers of cases.

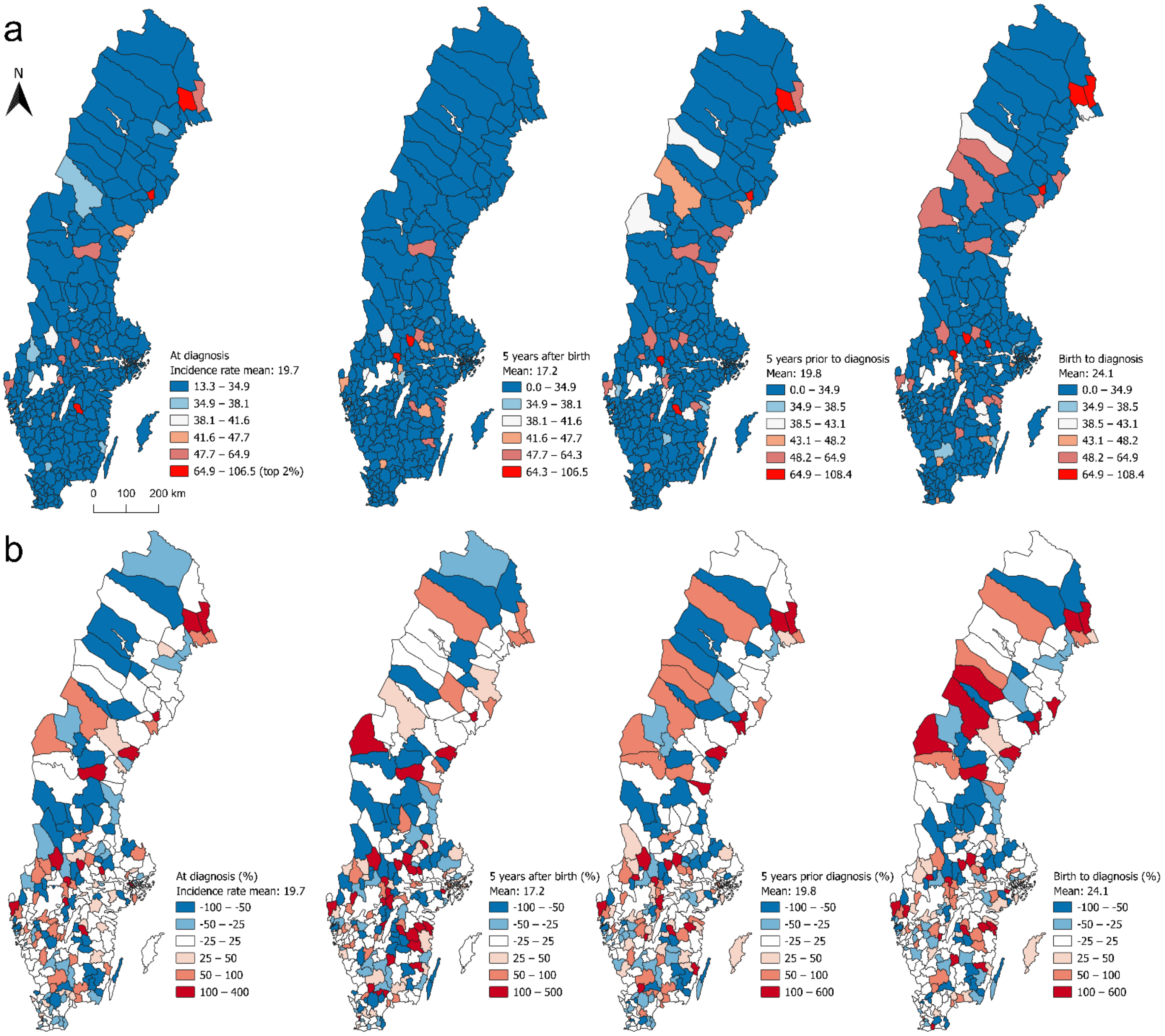

ESM Fig. 2: Geographical distribution at the municipality level for four different life stage-specific exposure windows of the incidence of type 1 diabetes in non-native individuals diagnosed in Sweden between 2005 and 2022 at the age of 0-30 years. a) Crude incidence of type 1 diabetes (cases per 100,000 person-years) by native/non-native background. Displayed in quintiles and the top 2% of the highest rates; b) Differences (%) between observed and expected numbers of cases.

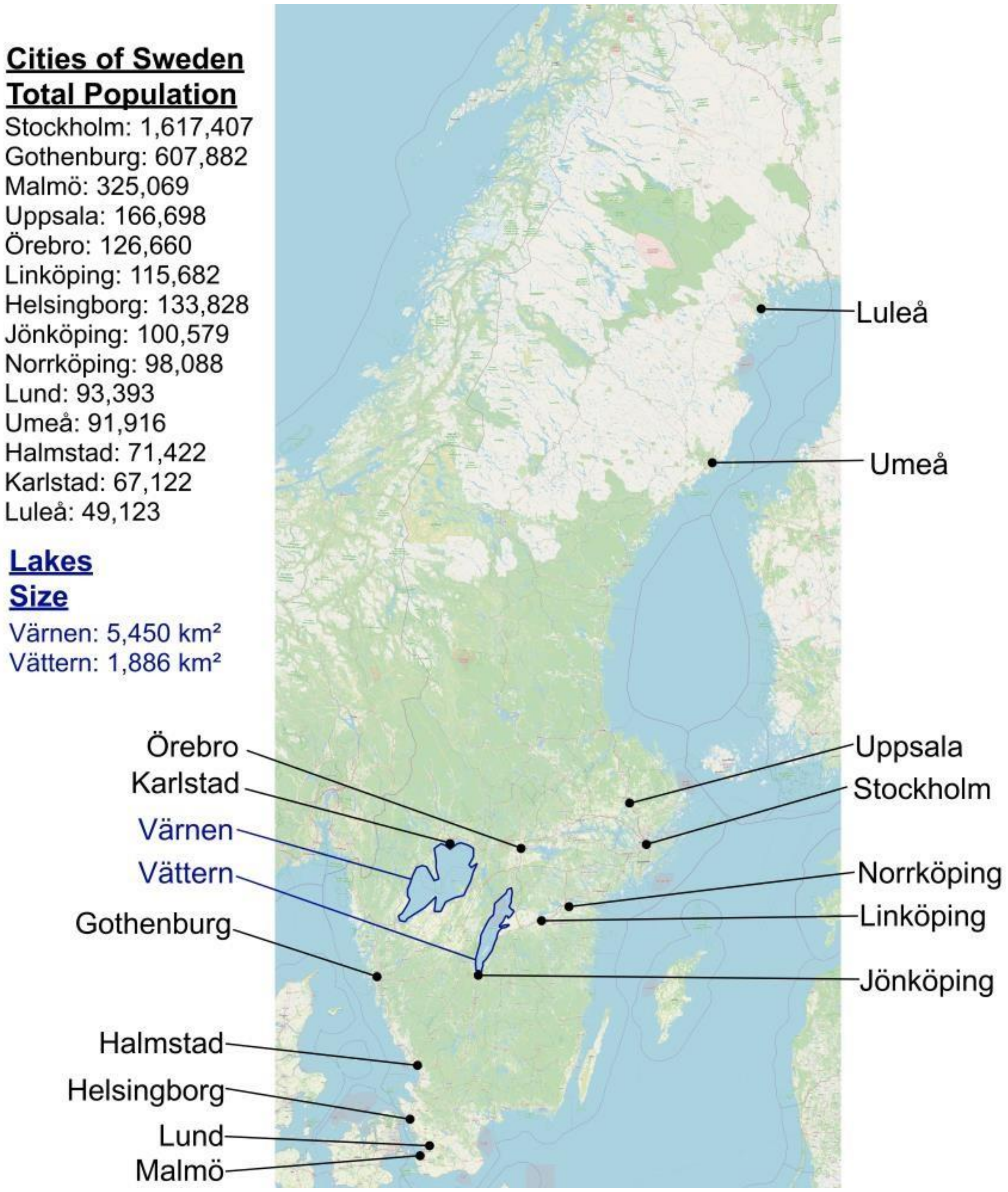

ESM Fig. 3: Map of Sweden, displaying most of the largest cities including all low-risk clusters, total population size and the two largest lakes.

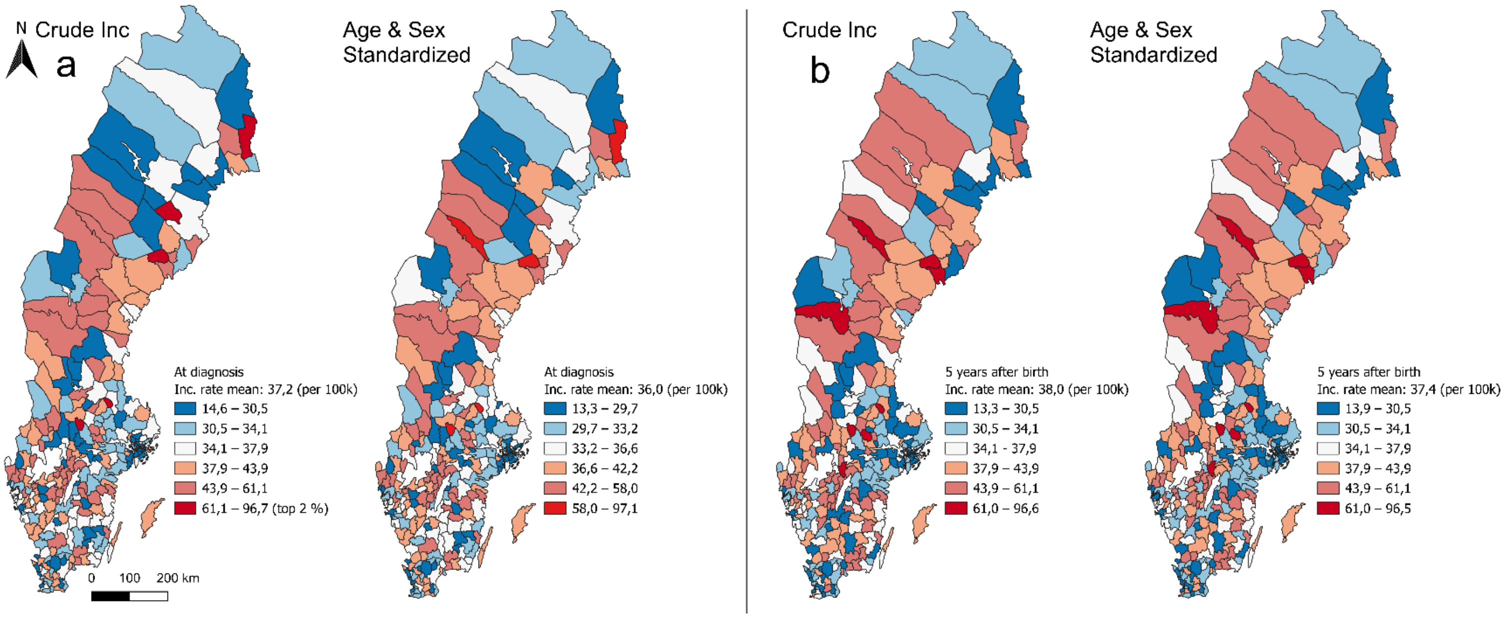

ESM Fig. 4: Geographical distribution at the municipality level for two different life stage-specific exposure windows of the incidence of type 1 diabetes in individuals diagnosed in Sweden between 2005 and 2022 at the age of 0-30 years. Displayed in quintiles and the top 2% of the highest rates. a) At diagnosis: Crude and standardized incidence of type 1 diabetes (cases per 100,000 person-years). b) 5 years after birth: Crude and standardized incidence of type 1 diabetes (cases per 100,000 person-years).
